# Deprivation and NHS General Ophthalmic Service sight testing activity in England in 2022–2023

**DOI:** 10.1111/opo.13399

**Published:** 2024-10-08

**Authors:** Robert A. Harper, Jeremy Hooper, David J. Parkins, Cecilia H. Fenerty, James Roach, Michael Bowen

**Affiliations:** ^1^ Manchester Royal Eye Hospital and Manchester Academic Health Sciences Centre Manchester University NHS Foundation Trust Manchester Manchester UK; ^2^ Division of Pharmacy and Optometry, School of Health Sciences, Faculty of Biology, Medicine and Health University of Manchester Manchester UK; ^3^ Conclusio, Cardiff Gate Business Park Cardiff UK; ^4^ Institute of Optometry London UK; ^5^ College of Optometrists London UK

**Keywords:** deprivation, primary care optometry, sight‐testing

## Abstract

**Purpose:**

Socioeconomic deprivation is associated with an increased incidence of sight‐loss. To inform potential developments in eyecare, General Ophthalmic Service (GOS) sight‐testing activity was explored in relation to deprivation for GOS contractors submitting National Health Service (NHS) claims in England.

**Methods:**

Data on NHS sight‐test claims for the financial year 2022–2023 were sought from NHS England (NHSE), including number of sight‐tests by GOS contractors, their unique Organisation Data Service codes and postcodes and age‐bands of patients accessing sight‐testing. Deprivation scores were assigned to contractor practices using the Index of Multiple Deprivation (IMD) and the average number of sight‐tests for all contractors within each IMD decile calculated, allowing rate of sight‐testing per 1000 population per decile of deprivation to be estimated using Office of National Statistics (ONS) Lower Layer Super Output Area mid‐year population estimates. Inequality was examined using the Odds Ratio (OR) and slope and relative index of inequality measures (SSI and RII).

**Results:**

Overall, 12.94 million NHS sight‐tests were provided by 5622 GOS contractors in England in 2022–2023. Most affluent decile GOS contractors undertook an average ~2200 NHS sight‐tests, while in the most deprived decile, average NHS sight‐tests per contractor was ~1100. Rate of sight‐testing per 1000 population in the most deprived decile was one quarter of that in the most affluent, with an OR of 5.29 (95% CI 5.27–5.30), indicating those in the most affluent areas were ~five times more likely to access NHS sight‐tests. Overall, SII and RII were 333.5 (95% CI 333.52–333.53) and 6.4 (95% CI 6.39–6.40), respectively, findings reflective of substantial inequality in uptake.

**Conclusion:**

There remains substantial unwarranted variation in uptake of NHS sight‐testing, with those in more affluent areas accessing sight‐testing substantially more than those in more deprived areas. Strategies are required to facilitate primary care optometry to provide more equitable access to eyecare.

**Key points**

- The association between socioeconomic deprivation and eye disease is well established, with previous research also highlighting those least deprived being more likely to access National Health Service funded sight‐tests than those more deprived.
- Investigating sight‐testing uptake in post‐pandemic England in 2022–2023, we find significant inequality remains, with those in the most affluent areas being more than five times more likely to access sight‐testing than those in the most deprived areas.
- UK National Health Service sight‐testing manifests significant unwarranted variation in uptake, amplifying previous calls for improvements to the eyecare system to reduce inequality, highlighting the pressing need for more research with publicly available datasets to inform such developments.

## INTRODUCTION

The association between socioeconomic deprivation and eye disease has long been recognised globally, with a systematic review identifying recurring factors, including low income and low educational attainment being associated with an increased incidence of sight‐threatening conditions.^1^ In the United Kingdom, research shows that socioeconomic deprivation continues to be associated with more advanced eye disease.^2^ National Health Service (NHS) sight‐tests under the General Ophthalmic Services (GOS) by optometrists in primary care as well as privately funded eye examinations remain the underpinning eyecare services via which most eye disease is detected and referred into secondary care ophthalmology. Primary care optometrists also provide wider NHS services, either as part of local commissioning, for example, Integrated Care Board commissioned enhanced or extended services in England, or within enhanced GOS models elsewhere. Small‐area analysis of GOS activity in Leeds found populations in the least deprived areas were more likely to have NHS funded sight‐tests than those in more deprived areas,^3^ with small‐area data modelling in Essex^4^ reinforcing the need to address inequalities. The overall number of NHS sight‐tests undertaken in England was ~9.16, 12.77 and 12.94 million for the years 2020–2021, 2021–2022 and 2022–2023, respectively, with the first of these periods being impacted by the COVID‐19 pandemic restrictions, resulting in fewer sight‐tests compared to subsequent years. We have recently published an analysis revealing substantial variations in the crude rate of optometric practices per 100,000 of population in England, with this rate being much lower in the more deprived versus more affluent areas, with some evidence for increased practice closures in the most deprived areas.^5^ However, updated evidence was not furnished around NHS sight‐testing activity for these GOS contractors. Given the earlier work of Shickle et al.^3^, ^4^ and with inequity of access to primary care optometry services seemingly being a significant unresolved concern in eyecare, we wanted to explore more recent post‐pandemic NHS sight‐testing activity in the GOS. The purpose of this paper is to explore sight‐testing activity per decile of deprivation for GOS contractor practices in England during 2022–2023, in the context of providing updated evidence for potential developments in eyecare aimed at reducing inequality.

## METHOD

Limited GOS sight testing data are freely available within the public domain^6^ and our initial data access request for more detailed GOS sight‐testing information was made to NHS Business Services Authority (NHSBSA) who referred the query to Primary Care Support England (PCSE). PCSE were advised by NHS England (NHSE) that due to there being no formal process in place to access these data, it would need to be the subject of an NHSE Freedom of Information (FoI) request. An initial FoI request was made in February 2024, with clarification requested in March 2024. GOS sight‐testing data were requested, including the financial year 2022–2023, for the following: the number of sight‐tests accepted for payment in England by GOS contractors; provision of GOS contractors' unique Organisation Data Service (ODS) codes and postcodes and numbers accessing sight‐testing from age categories including children, working age adults and adults ≥65 years of age.

Data were made available for analyses by NHSE in Microsoft Excel (Microsoft.com) file format. There were no information governance restrictions by NHSE England as no patient identifiable data were provided, as defined within the Health and Social Care Act 2012. A deprivation score was assigned to the location of each contractor practice using the Index of Multiple Deprivation (IMD) 2019.^7^ The average number of sight‐tests for contractors was calculated within each deprivation decile. Next, the rate of sight‐tests per 1000 population was calculated for each IMD decile using Office of National Statistics (ONS) Lower Layer Super Output Area (LSOA) mid‐year population estimates.^8^

Measuring inequality has complexity, and a number of options were considered, using the Technical Guide from the Office for Health Improvement and Disparities^9^ and the Scottish Public Health Observatory Approaches to measuring health inequality^10^ to inform our choice. Three different measures were chosen to quantify inequality in the uptake of sight‐testing, potentially helpful for considering strategies to reduce inequality in both eye and wider health outcomes. The measures used included: the odds ratio (OR); the Slope Index of Inequality (SII) and the Relative Index of Inequality (RII). SII measures the absolute inequality in sight‐testing, for example between the most and the least affluent areas, while RII measures the ratio between sight‐testing in the most affluent and the least affluent areas. The OR provides a simple assessment of the greater number of sight‐tests undertaken in the more affluent areas relative to the more deprived areas, whereas the SII and RII measures are regression based and therefore consider the whole population.

## RESULTS

### Rate and number of sight tests by GOS contractors by deprivation decile

**FIGURE 1:**
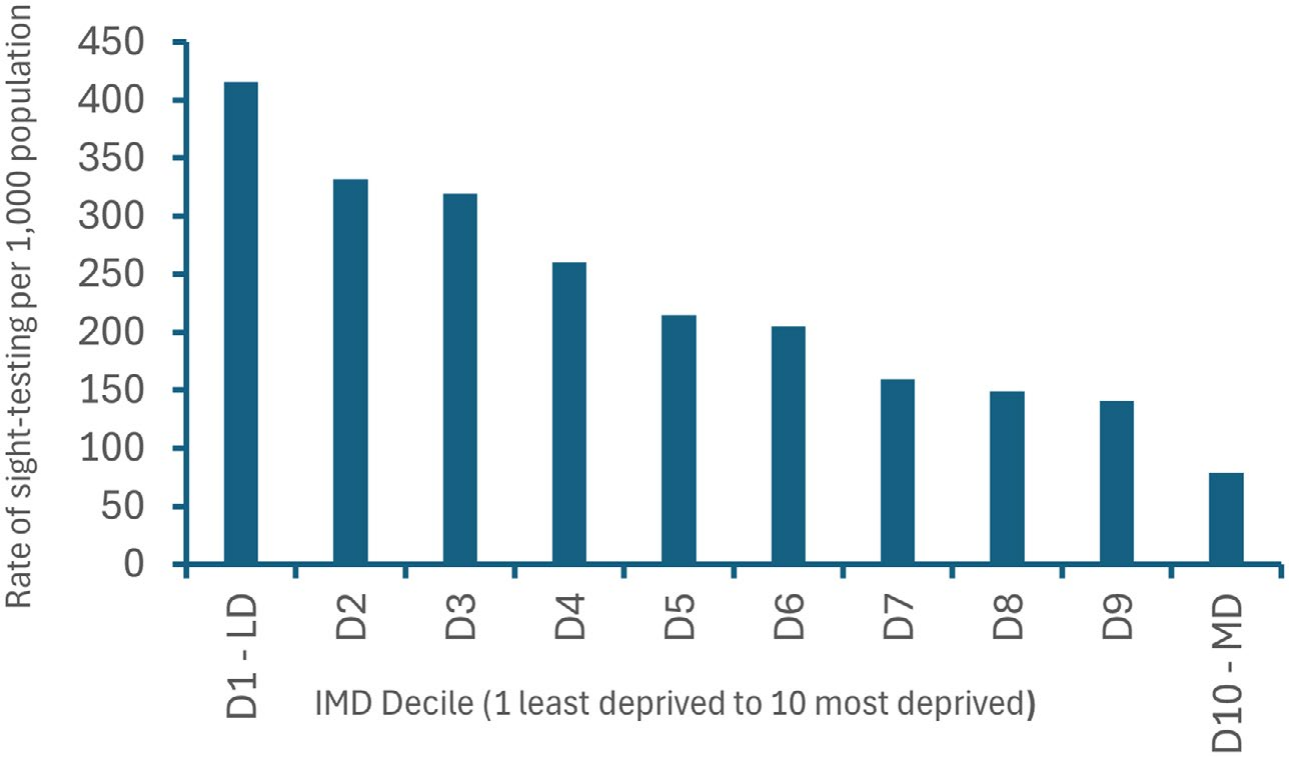
Rate of National Health Service (NHS) sight‐tests in 2022–2023 per 1000 of the population by General Ophthalmic Services (GOS) contractors in each Index of Multiple Deprivation (IMD) (2019) decile (D1–D10) from the least deprived (LD, far left) to the most deprived (MD, far right). The 95% confidence intervals were calculated for these data using Byar's method, with confidence intervals being extremely tight (and therefore not illustrated) owing to very large sample sizes, ranging from ±0.003 to ±0.001, indicating each decile is significantly different to all other deciles.

**FIGURE 2:**
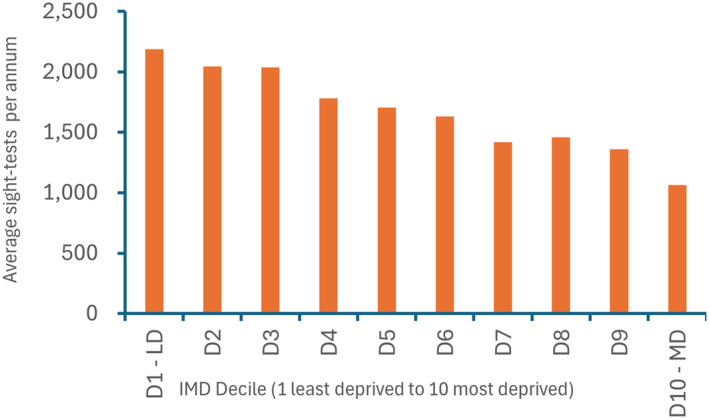
Average number of National Health Service (NHS) sight‐tests by General Ophthalmic Services (GOS) contractors in 2022–2023 in each deprivation decile during 2022–2023, with providers in decile 1 (D1), the least deprived (LD) far left, undertaking almost twice as many sight‐tests compared to providers in decile 10 (D10), the most deprived (MD) decile. Note, 95% confidence intervals using Byar's method are very tight indeed (and therefore not illustrated) owing to very large sample sizes, with confidence intervals of ~±0.02 for all deciles, indicative that each decile is significantly different to all other deciles.

Overall, the 12,941,325 NHS sight‐tests for 2022–2023 were provided by 5622 GOS contractors in England, including domiciliary sight‐tests. In considering the impact of deprivation, the average rate of sight‐testing by GOS contractors per 1000 population was estimated in each decile of deprivation (i.e., based on the IMD decile of the GOS contractor practices), illustrated in Figure 1. In the most deprived decile, the rate of sight‐testing was approximately one quarter of that seen in the most affluent decile; a finding likely reflecting both a lack of uptake in more deprived areas but also fewer (and potentially smaller) practices in these more deprived areas. To understand the uptake of sight‐tests across IMD deciles, Figure 2 shows the average number of sight‐tests undertaken by GOS contractors within each deprivation decile. In the most affluent decile, contractors undertook an average of ~2200 NHS sight‐tests in 2022–2023, while in the most deprived decile in the same year the average number of sight‐tests per contractor was half this figure, at ~1100 sight‐tests.

### Inequality analysis

The OR was calculated by dividing the rate of sight‐testing in the most affluent decile (D1) by the rate of sight‐testing in the most deprived decile (D10). A relative OR of 5.29 (95% CI 5.27–5.30) is estimated, suggesting that people accessing sight‐testing in the most affluent areas are over five times more likely to have sight‐tests. SII and RII measures were calculated using rates per 1000 population as described earlier. Both these indices of inequality should equate to zero if there was an absence of inequality. For overall national data in 2022–2023, SII was 333.52 (95% CI 333.52–333.53) and RII was 6.40 (95% CI 6.39–6.40), with both indices showing substantial inequality in sight‐testing uptake, with patients in the least deprived areas accessing GOS services substantially more than those in the most deprived areas.

### Sight testing by age

**FIGURE 3:**
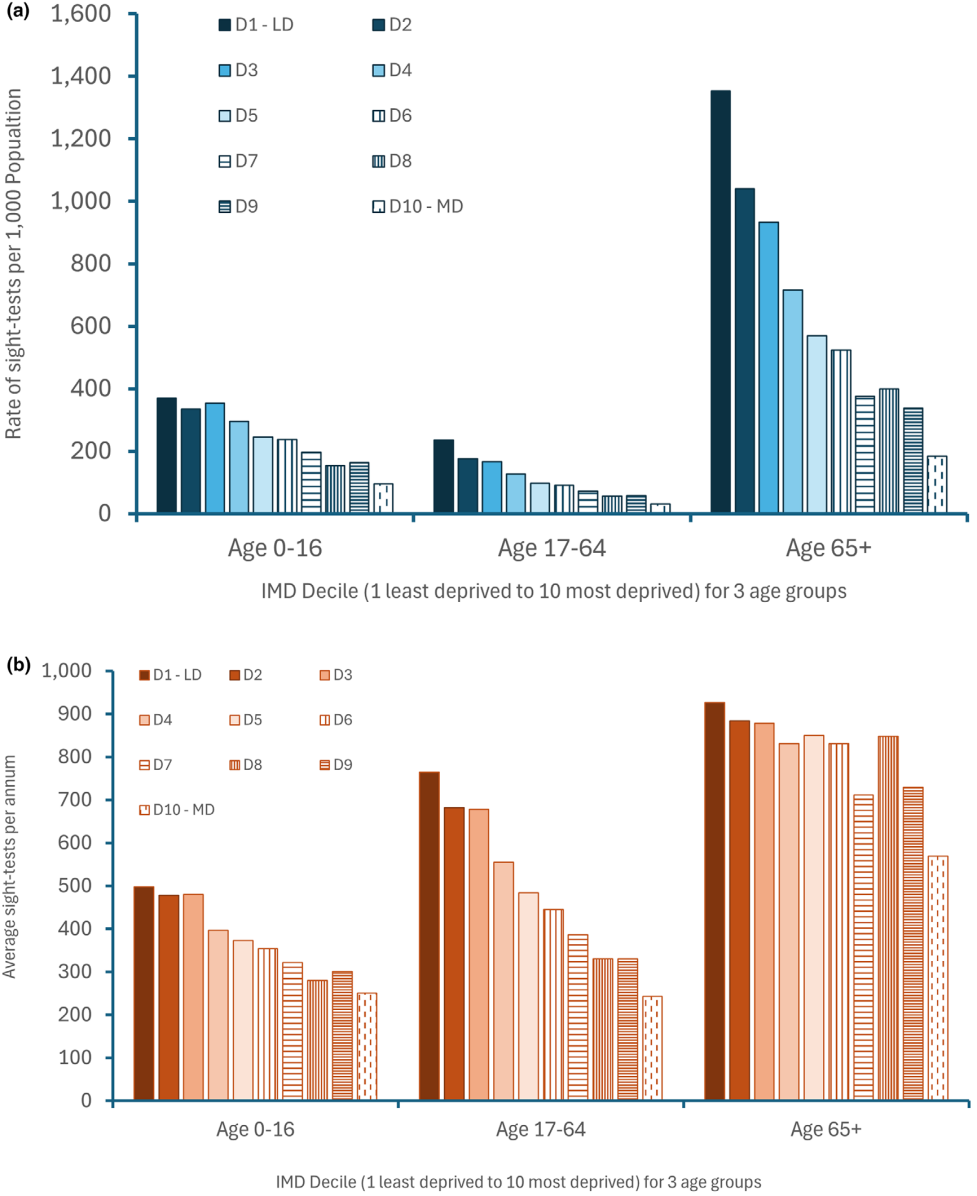
The average rate per 1000 population (a, top) and number of National Health Service (NHS) sight‐tests (b, bottom) undertaken by General Ophthalmic Services (GOS) contractors within each deprivation decile across three age categories in 2022–2023. Overall, the rate of NHS sight‐tests (top) was greatest for those aged 65 years of age and over, and lowest for those aged 17–64 years, reflective of NHS eligibility. IMD, index of multiple deprivation.

Analysis for the three age categories (which differ slightly from GOS sight‐testing eligibility categories) showed a marked difference in the number of tests undertaken. Figure 3 illustrates the average number of NHS sight‐tests undertaken by GOS contractors within each IMD decile. Interestingly, the least deprived two deciles (i.e., contractors in affluent areas) show a rate of sight‐testing >1000 per 1000 population for those 65 years and over, consistent with greater sight‐testing per contractor in these affluent locations; areas where it is known there are more contractors and likely larger practices providing services. In terms of disparities by deprivation decile, inequality analyses by age‐categories (summarised for the rate of sight‐testing in Table 1) replicates the position shown in overall national sight‐testing analyses, with higher rates of sight‐testing in least deprived deciles and lower rates in more deprived deciles. The OR was smallest in the 0–16 years of age category at 3.89 (95% CI 3.87–3.92), with the OR being similar in the other age categories, that is, OR of 7.57 (95% CI 7.52–7.62) for ages 17–64 years and OR of 7.36 (95% CI 7.34–7.39) for those aged 65 and over. This finding suggests that inequality is lower in children in this specific context, although children in more affluent areas are still over three times more likely to have a sight‐test than those in more deprived areas. In adults, those in more affluent areas were over seven times more likely to have NHS sight‐tests in 2022–2023 than those in more deprived areas. RII was smallest (i.e., least inequality) in the 0–16 years of age category (4.02), with RII being similar in the other categories, that is, 17–64 years (RII 19.29) and the 65 years and over group (RII 19.54). For children, the gradient reflects that seen overall, with the average number of sight‐tests undertaken in the least deprived area being approximately double that seen in the most deprived areas. Within the over 65 year age‐group, the gradient from most to least deprived was less than that seen in the overall population, although the average number of sight‐tests reduces across deciles until the most deprived decile when it reduces substantially. The greatest disparity was seen in the working age category (where the proportion of NHS funded sight‐tests was smaller, compared to other age categories), with the steep gradient reflecting the average number of sight‐tests being ~three times greater in least compared to most deprived areas.

**TABLE 1:** SII, RII and OR (and companion lower and upper 95% confidence limits, CL) for the three age categories within 2022–2023 GOS NHS sight testing data, showing substantial inequality in the rate of sight testing per 1000 population between the least and most deprived deciles.

Age band (years)	Slope index of inequality (lower and upper 95% CL)	Relative index of inequality (lower and upper 95% CL)	Odds ratio (lower and upper 95% CL)
0–16	304.49 (304.49–304.50)	4.02 (4.02–4.02)	3.89 (3.87–3.92)
17–64	204.30 (204.29–204.30)	19.29 (19.29–19.29)	7.59 (7.52–7.62)
≥65	1052.20 (1052.18–1052.21	19.54 (19.54–19.54)	7.36 (7.34–7.39)

Abbreviations: GOS, General Ophthalmic Services; NHS, National Health Service; OR, odds ratio; RII, Relative Index of Inequality; SII, Slope Index of Inequality.

## DISCUSSION

There is a stark difference in the number of NHS sight‐tests and the rate of sight‐testing per decile of deprivation by GOS contractors operating in England during 2022–2023, with NHS services preferentially being accessed by patients attending contractors in more affluent areas. We have previously observed that primary care optometry contractors in England are inequitably distributed in relation to deprivation.^5^ In the present analyses, it is apparent that there are almost twice as many NHS sight‐tests being undertaken by contractors in the least compared to the most deprived areas, with the rate of sight‐testing in the most deprived decile being approximately one quarter of that seen in the most affluent decile. These data reflect recent unwarranted quantitative evidence for significant inequality in the uptake of sight‐testing between affluent and deprived areas, with a consistent and significant pattern across deciles of deprivation.

The rate of sight‐testing in areas of more affluence being at a rate of >1000 sight‐tests per 1000 population in the over 65 years of age category merits further comment. This finding is likely reflective, in part, of patients' travel away from residential localities to seek eyecare and/or competitive pricing (i.e., a move to more affluent areas for sight‐testing, locations where there are more practices available compared to deprived areas). It is acknowledged that the funding model for primary care optometry relies on the cross‐subsidisation of GOS sight‐testing (which incorporates eye health examinations) by the sale of spectacles, linking with services being situated where most economically viable. In turn, this acknowledgement coexists with concern about costs and the perceived pressure to buy spectacles for some.^11^ There is also potential for greater numbers having shorter testing intervals when deemed clinically necessary (and therefore double counting within the year) in the case of the more elderly patients, but this possible contributor appears less impactful in the more deprived deciles. Indeed, shorter testing intervals may impact all deciles to some extent. More broadly, risk factors for eye disease conferring GOS sight‐testing eligibility may also be more prevalent in deprived areas, for example, the higher prevalence of diabetes linked to socioeconomic deprivation,^12^ thereby amplifying concern about the potential impact of a differential uptake in sight‐testing and/or other eyecare services.

Limitations to analyses of these data merit discussion. First, these analyses reflected only GOS NHS sight‐testing data versus data also including private eye examinations, although the former reflects in excess of 70% of all primary care eye examinations, while private eye examinations may be argued to be more reflective of affluent area activity. Second, the NHS sight‐testing rate estimated here is simply the number of sight‐tests undertaken in each IMD decile divided by the population of each decile. We are not able to comment on populations of patients served by contractors per se. We acknowledge that a limitation of these data is that we do not know where the individuals accessing sight‐testing reside in relation to the contractors. For example, claims for domiciliary sight‐tests may be processed through a head office location, and not the area within which sight‐testing was provided. Further, allocation of one IMD score per contractor is highly unlikely to reflect the whole population served by the contractor, which likely has patients with different IMD levels, an acknowledgement of the well‐reported complexity of socioeconomic status, neighbourhood deprivation and health.^13^ Considering deprivation at the GOS contractor level versus that of patients accessing NHS sight‐testing is likely to mean these analyses are unlikely to capture the impact of local neighbourhood deprivation, with IMD values in high streets not necessarily reflecting the neighbourhood in which they are situated. Further, data are not available regarding eligibility for NHS sight‐testing criteria beyond age, so for example within the 17–64 years of age group, their reason for eligibility may be income related or due to the presence of diabetes, glaucoma or risk of glaucoma. Previous local area studies using 2011 data^3^ and 2013–2015 data^4^ found higher GOS uptake in the more deprived quintiles among 16–59 year olds due to means tested social benefits. These 2022–2023 data for England do not show this effect. Factors such as high inflation and the increased cost of living may be possible reasons why people in a similar age group were not accessing NHS sight‐tests, albeit the data are England‐wide, and therefore may not show the variation observed at the regional, system and place levels. Further research and open access to data are required at the system and place levels to better understand such differences and local variations. It is also unclear what the impact of patients' home and/or work locations and their access to private or public transport (and associated costs) might have in relation to these data, since it is known, for example, that distance to practitioners is a perceived barrier to the uptake of eyecare. Third, it may be argued that while these ‘big data’ reflect unbiased large numbers with very tight confidence intervals, there may be unknown limitations owing to collation errors within the dataset being interrogated. Finally, due to changes in geography following the 2021 census and because the deprivation data have not been updated to reflect such changes, it was necessary to use the 2020 LSOA mid‐year population estimates from ONS. The IMD score was updated in 2019 and while this version may be outdated, despite limitations, IMD remains the best readily available method for examining deprivation. We believe that the various limitations discussed here are unlikely to have an effect that unpicks the stark major trends observed.

There is longstanding recognition of the importance of the impact of deprivation in eyecare, but no real evidence concerns conveyed previously are resulting in plans to change the system for primary eyecare. Data such as those highlighted here may support policy makers to target finite NHS resources to those most in need, versus supporting a system favouring affluence. Research should be facilitated by healthcare datasets being made freely available, facilitating ongoing evaluation of publicly funded services, thereby allowing commissioners to use data to ensure equity of access to optometry services. Indeed, one outcome of this research is to call for better arrangements for the sharing of data with researchers, and if place‐of‐residence data could be recorded within an agreed range that satisfied pseudonymisation but allowed enough detail to relate to IMD locations, such a step would increase the scope for research to explore inequalities in the uptake of services. Nevertheless, and based on what is already known, Shickle et al.^14^ concluded that the GOS contract may be contrary to public health interests, proposing that different approaches are needed to address eye health inequalities to reduce preventable sight loss. A decade on, these data appear to reinforce a still outstanding need for change.

## AUTHOR CONTRIBUTIONS

**Robert A. Harper:** Conceptualization (equal); data curation (supporting); formal analysis (supporting); methodology (equal); project administration (equal); writing – original draft (lead); writing – review and editing (lead). **Jeremy Hooper:** Conceptualization (equal); data curation (lead); formal analysis (lead); project administration (equal); software (lead); writing – original draft (supporting); writing – review and editing (supporting). **David J. Parkins:** Conceptualization (supporting); methodology (supporting); writing – original draft (supporting); writing – review and editing (supporting). **Cecilia H. Fenerty:** Conceptualization (equal); data curation (supporting); formal analysis (supporting); methodology (supporting); writing – original draft (supporting); writing – review and editing (supporting). **James Roach:** Conceptualization (equal); data curation (supporting); methodology (supporting); project administration (supporting); software (supporting); writing – original draft (supporting); writing – review and editing (supporting). **Michael Bowen:** Conceptualization (supporting); methodology (supporting); writing – original draft (supporting); writing – review and editing (supporting).

## FUNDING INFORMATION

There was no funding for this research.

## CONFLICT OF INTEREST STATEMENT

Robert Harper and David Parkins are Life Fellows of the College of Optometrists where Michael Bowen is Director of research. David Parkins has an advisory role at NHSE London.
